# Mutations in *C-KIT* exon 11 in canine cutaneous mast cell tumors

**DOI:** 10.1186/1753-6561-7-S2-P56

**Published:** 2013-04-04

**Authors:** Rafael T Neto, Didir Q Cagnini, Renée L Amorim

**Affiliations:** 1 Department of Clinical Veterinary Medicine, School of Veterinary Medicine and Animal Sciences, Universidade Estadual Paulista (UNESP), Botucatu, SP, Brazil

## Background

The *c-KIT* proto-oncogene encodes the receptor tyrosine kinase KIT, which has been shown to play important roles in the cellular maturation, survival, proliferation, and migration of several cell types including mast cells. Mast cell tumors (MCTs) are the most common cutaneous tumor in the dog. MCTs exhibit wide variation in biological behavior. *KIT* mutations and aberrant KIT expression have been identified in canine MCTs. Unlike human mastocytosis patients, in which point mutations primarily occur in the kinase domain of KIT, internal tandem duplications (ITD) have been identified in the juxtamembrane domain of *KIT* in canine MCTs. Among several *KIT* mutations identified, an ITD in exon 11 has been analyzed most consistently and is significantly associated with malignant behaviour of affected tumors. The goal of this pilot study was to describe ITD *KIT* mutation in six high grade MTCs and four low grade MCTs.

## Materials and methods

PCR amplification mutational analysis of *KIT* was performed using a primer pair for *C-KIT* exon 11, forward primer 5^’-^CCATGTATGAAGTACAGTGGAAG-3’ and reverse primer 5’-GTTCCCTAAAGTCATTGTTACACG-3’. PCR products were analyzed by 1.5% agarose gel electrophoresis and visualized with SYBR^®^ Safe DNA Gel Stain. Two of six high grade MCTs (2/6) had ITD *KIT* mutations. All of these tumors had aberrant diffuse cytoplasmic c-KIT (CD117) localization by immunohistochemistry.

## Results and conclusions

None of low grade tumors 4/4 had ITD *KIT* mutations, and of these tumors only one (1/4) had aberrant c-KIT immunostaining. Internal tandem duplication of *KIT* exon 11 mutation was associated with high grade tumors and aberrant C-KIT localization by immunohistochemistry in canine MCTs.

## Financial support

FAPESP.

